# The Mortality of Child-Bearing

**Published:** 1919-06-14

**Authors:** 


### THE MORTALITY OF CHILD-BEARING.

### Reasons for Continued High Figures.

In the paper read a short time ago before the Obstetric Section of the Royal Society of Medicine by Victor Bonney, M.S., F.R.C.S., many facts of the greatest interest to both the profession and health workers were brought to light. Unfortunately, the review is by no means favourable to previously accepted measures, but we are nevertheless indebted to the author for an essentially constructive criticism.

In the first place, to refer to figures alone, one notes that according to registrarial returns for England, Wales, and Scotland, the death-rate directly and indirectly attaching to pregnancy and labour does not markedly differ from what it was seventy years ago. In 1915 a report stated that the number of registered births corresponding to one maternal death was, in England 250, in Wales 179, and in Scotland 175. Also, according to American findings, out of a selection of the most accessible nations only England and Wales, Ireland, Japan, New Zealand, and Switzerland have recorded any lessening in child-bearing mortality during recent years, and of these, it is only in the British Isles that they show any reduction in the number of fatal cases of puerperal septicæmia. Even then sepsis is said to account for about 30 per cent. of the total deaths!

Although in this country we might derive some poor satisfaction from the fact that we are at the right end of the list, yet it is obvious that such records, viewed generally, prove that the obstetrics of the whole world must be singularly at fault. For no improvement in results to have occurred over a period of years when every other branch of the medical profession has advanced by leaps and bounds, suggests that something must be radically wrong with our procedure, but Mr. Bonney finds fault with both the public and professional conception of midwifery rather than with modern technique as exemplified at our hospitals.

### The Absence of "Surgicalness."

It is an unfortunate fact that midwifery is not generally and strictly established as a department of surgery, and that ordinary obstetric procedures too frequently take place under conditions which must appal men of good surgical training. Why should it not be considered essential to convert the confinement room into a condition bearing some semblance to an aseptic operating-theatre, and to have the skilled assistance of an independent anæsthetist? We know that in good-class practice these matters are to some extent attended to, but it is certainly not the case with the great majority of births occurring in this country. Seldom do we see the need for such refinements urged, yet surely the profession cannot consider them as institutional ideals not to be hoped for in everyday work. Leaving aside the well-known awfulness of surroundings under which obstetrics are performed in the poorer districts, the author reminds us that the absence of "surgicalness" is almost everywhere prevalent.

If we remember that reproduction *is* a physiological process, but stands alone in that it is not exercised on behalf of the mother, but on behalf of the race—and at the expense of the mother—and also that the product of conception is as truly a neoplasm as any other growth within the uterus; that labour is a true surgical operation which the woman *herself* performs, then the general failure to recognise the root cause of past failures becomes clearer. The product of conception which, as everyone knows, can cause hepatic or renal disease, convulsions, hæmorrhage, or pelvic impaction is no less a life-endangering neoplasm than any of the tumours met with in gynæcological practice. Its treatment is strictly surgical, and that of the most advanced type, where, apart from actual technique, precautions are taken that abnormal pregnancy shall not escape medical supervision until it has advanced too far, or that the discovery being made, there is no delay in applying the correct surgical principle that a life-menacing tumour should be got rid of forthwith. In any event, "labour, even normal labour, should be considered as an operation. The first requisite for safety, therefore, is asepsis of the operation area, or birth area, as we will call it. The vagina should be regarded as a wound into which the passage of anything in a fumbling, half-sighted manner and without previous antiseptic preparation of the surrounding skin is a hideous transgression of the ritual of modern aseptic surgery. And under the term obstetric operation . . . include not the obviously mechanical procedures, such as forceps extraction, craniotomy, and so on, but every manual assistance to delivery, even if it run to no more than the hooking down of an arm, a single stitch in the perineum, or a vaginal examination."

### Some Fallacies.

In spite of the increasingly frequent use of rubber gloves in obstetrics—a partial prevention from extrinsic infection—it stands to reason that the organisms of the patient's skin, particularly of the ano-perineal region, are transferred equally readily by the gloved as by the ungloved hand. It is perhaps not too much to say that in the overwhelming majority of instances, it is organisms from this source which are the chief agents of puerperal sepsis. Therefore it becomes of paramount importance completely to sterilise the birth area.

This by many has been viewed as an impossible task, perhaps not worth attempting under conditions of urgency, but we may quote Mr. Bonney in an attempt to persuade the sceptics:

> The investigations carried out by Dr. C. Browning and myself showed that sterilisation of the ano-perineal region could be effected by the use of "violet-green," and I have suggested that during labour this antiseptic should be applied by compress to the vulvo-perineal skin until such time as the head is about to be born. Further, I think that this antiseptic should be used every time a vaginal examination is made. . . .

These antiseptics, "violet-green," "flavine," etc., are extremely powerful germicides, can be

**The Mortality of Child-Bearing**—(*continued*).

freely used without fear of irritation to skin or mucous membrance, and in sufficient strength to go very far towards producing efficient sterilisation. The staining of either maternal or fœtal parts is a small price to pay if this end be reached. But in practice probably greater carelessness is displayed in maintaining than in attaining asepsis, and yet the former is the simpler problem. We have but to copy the ordinary arrangements of a modern operating-theatre. The lying-in chamber should be prepared strictly according to these ideals. All maternity nurses should be thus instructed, yet, at the present time, not one in fifty makes a satisfactory attempt. Partly is this due to want of teaching, partly to the ignorance of the patient and her relatives, who insist on the bedroom trumpery. It is all a matter of education.

To both the doctor and the relatives Mr. Bonney points out that, at a cost of less than a sovereign, a tin containing a complete outfit of sterilised gowns, towels, swabs, and gauze can now be obtained. Without such an outfit the reasonably aseptic conduct of labour cannot be performed, and the layman, niggardly of all expenditure where childbirth is concerned, must be made to realise that no money could ever be better spent.

The Third Stage.

There is an exact parallel between the wound left in the uterus after the vaginal enucleation of a fibroid and that left by the separation of the placenta; also between the perineal tear and that free surface produced in plastic enlargement of the vaginal orifice. Their treatment must be identical. Again, the practice of exploring or douching the uterine cavity is stated to be far less frequently necessary than is customarily taught. Many mistaken ideas of the value of such procedure are perpetuated by the erroneous teaching that puerperal septicæmia is commonly *caused* by gross portions of the placenta retained in the uterus, whereas such is certainly not the usual sequence. Unfortunately to our means of treating puerperal sepsis it is impossible more than partially to apply the new methods so successfully adopted with bullet and shell wounds. Mainly must we use prevention as our only weapon against it.

Public Custom and the Doctor.

It is unimaginable even to the public that a surgeon should perform a curettage or ligature piles without an anæsthetist to assist him, but even to this day, in obstetric work, the practitioner is often diffident of asking for such aid, since an amazingly unenlightened laity expects him to combine the offices. Even the practice of anæsthetic administration by the nurse is to be condemned utterly, for all such compromises are irreconcilable with an aseptic technique, while in a case of difficulty or emergency the result is hopeless chaos. Also it is impossible for the obstetrician to guard against contamination, gloved though his hands may be, unless he has, besides the anæsthetist, a skilled assistant at his side. In the present state of affairs lack of public appreciation of the refinements of his art has resulted in the habitual underpaying of the obstetrician, though the skill, equipment, and time demanded from him should be at least as great as in other branches of surgery. The passing of the Midwives Act and the recent establishment of ante-natal clinics in various parts of the country have made progress towards improvement, but still we are far from the ideal. A midwife, even less than a doctor, does not, single-handed, comply even approximately with the requirements of labour, and this the public and profession alike must realise before the disappointing figures given earlier can be lastingly bettered.

Some Remedies.

As Mr. Bonney suggests, it is a fact that to the doctor midwifery does not pay, and under-paid work cannot be the best work.

> On the other hand, it is essential that the monetary cost of child-bearing . . . should not be so high as to discourage reproduction. It may be with much justice argued that the expenses of child-birth up to a certain equitable figure should in all cases be borne by the nation, to whose advantage the child is brought into the world.

As the public realise the value of full surgical technique, there will probably arise large lying-in hospitals all over the country, having private wards but maintained or partially maintained out of public funds. These hospitals should also be the centres of the teaching of midwifery, and extern departments as at present carried on must be abolished. Of the extern systems Mr. Bonney says:

> They perpetuate all the worst features of midwifery as practised to-day, the inadequate surroundings, the wretched light, the meagre assistance, and the dirt lead the student to think that the régime of the labour ward is an ideal unrealisable in general practice.

Two classes will be found among those women unable or unwilling to enter such institutions. The rich, who can avail themselves of nursing homes or private advantages, and the very poor, whose entry is impossible on account of domestic reasons or the sudden onset of unexpected labour. The latter might be dealt with by an extern "team"—an obstetrician, an anæsthetist, and two nurses—but not by one man as at present. Complete outfit, conveyance, and means to convert the meanest dwelling-room to cleanliness would be provided, and although local medical men would be urged to do all possible themselves, yet the teams or their component parts would be ready for immediate activity. Patients taking private rooms should be attended by their own doctor and pay him adequate fees. The permanent resident staff would assist him if necessary in the conduct of the labour.

Such is a rough sketch of the author's description of what is required before we can hope to see a progressive elimination of late complications, sepsis, and therefore in the mortality of child-bearing comparable with that already attained in practically every other recognised surgical procedure. Mr. Bonney is emphatic that midwifery must not necessarily be more "operative," but for it to be more "surgical" is essential. It must be taught and practised as a branch of surgery.

